# Amoxicillin-induced linear IgA bullous dermatosis mimicking erythema multiforme: a case report

**DOI:** 10.1093/skinhd/vzae024

**Published:** 2025-02-25

**Authors:** Marion Silagy, Priscille Carvalho, Billal Tedbirt, Clémence Tamarit, Marion Carrette, Florence Tétart, Alexis Lefebvre

**Affiliations:** Department of Dermatology, Rouen University Hospital, Rouen, France; Department of Dermatology, Rouen University Hospital, Rouen, France; Department of Dermatology, Rouen University Hospital, Rouen, France; Department of Anatomical Pathology, Rouen University Hospital, Rouen, France; Immunology Laboratory, Rouen University Hospital, Rouen, France; Department of Dermatology, Rouen University Hospital, Rouen, France; Department of Dermatology, Rouen University Hospital, Rouen, France

## Abstract

A 77-year-old man presented with a cutaneous rash of 3 days’ duration. Seven days before onset, the patient reported a bronchopulmonary infection treated with amoxicillin. Physical examination revealed multiforme cutaneous lesions, involving the armpits, pubis, genitals and lower back. In the lower back area, lesions were erythematous, purplish targetoid-like with multiple concentric circles. In places, bullae and postblistering erosions could be seen. In places, a ‘string of pearls’ pattern could be observed. Nikolsky sign was negative. Herpes simplex virus polymerase chain reaction (PCR) on mucosal erosions was negative. Multiplex nasopharyngeal PCR was negative for influenza virus, COVID-19 and *Mycoplasma pneumoniae*. Histopathological examination revealed spontaneous subepithelial cleavage with neutrophilic ­microabscesses. Direct immunofluorescence showed linear IgA deposition at the dermal–epidermal junction, confirming the diagnosis of linear IgA bullous dermatosis. Skin lesions were treated with topical clobetasol propionate cream and oral mucosa with corticosteroid mouth rinses. The disease course was marked by complete remission 7 days after amoxicillin discontinuation. There was no relapse after 4 months of follow-up.

**What is already known about this topic?**

- Linear IgA bullous dermatosis (LABD) is a rare subepithelial autoimmune disease, characterized by a linear deposit of IgA on the basement membrane zone.
- LABD can be idiopathic or caused by triggering factors such as drugs.
- To date, there is no clinical difference between idiopathic and drug-induced LABD.

**What this study adds**

- This case illustrates the polymorphism of drug-induced LABD.
- Skin biopsy for histopathological and direct immunofluorescence examinations should always be performed when bullous lesions are present.

##  

A 77-year-old man presented with a cutaneous rash of 3 days’ duration. His medical history included hypertension treated with irbesartan and bisoprolol, and Graves disease treated with carbimazole. He had no dermatological history. Seven days before onset, the patient reported a broncho­pulmonary infection treated with amoxicillin. The patient was hospitalized in the dermatology clinic for further examination and monitoring. Physical examination revealed multi­forme cutaneous lesions involving the armpits, pubis, genitals and lower back. On the lower back area, lesions were erythematous, purplish targetoid-like with multiple concentric circles. In places, bullae and postblistering erosions could be seen (Figure 1). Examination of the armpit area showed bilateral and symmetrical confluent annular lesions with a purpuric centre. In places, a ‘string of pearls’ pattern could be observed (Figure 2). Nikolsky sign was negative. Skin lesions provoked a painful burning sensation. Oral mucosa was also involved with small erosive lesions. Genital and anal mucosa were spared. Ophtalmological and nasopharyngeal examination found no mucosal involvement. Herpes simplex virus polymerase chain reaction (PCR) on mucosal erosions was negative. Radiological investigations found no sign of active pulmonary infection. Multiplex nasopharyngeal PCR was negative for influenza virus, COVID-19 and *Mycoplasma pneumoniae*. Histopathological examination revealed spontaneous subepithelial cleavage with neutrophilic microabscesses (Figure 3a). Direct immunofluorescence (DIF) showed linear IgA deposition at the dermal–epidermal junction, confirming the diagnosis of linear IgA bullous dermatosis (LABD) (Figure 3b). IgA salt-split skin indirect immunofluorescence and IgA immunoblotting using LAD1 were negative. Skin lesions were treated with topical clobetasol propionate cream, and the oral mucosa with corticosteroid mouth rinses. No systemic treatment was used. The disease course was marked by complete remission 7 days after amoxicillin discontinuation (Figure 4a,b). There was no relapse after 4 months of follow-up. Given a Naranjo score of 4, this skin manifestation was considered to be ‘possible adverse drug reaction’.^1^

**Figure 1:**
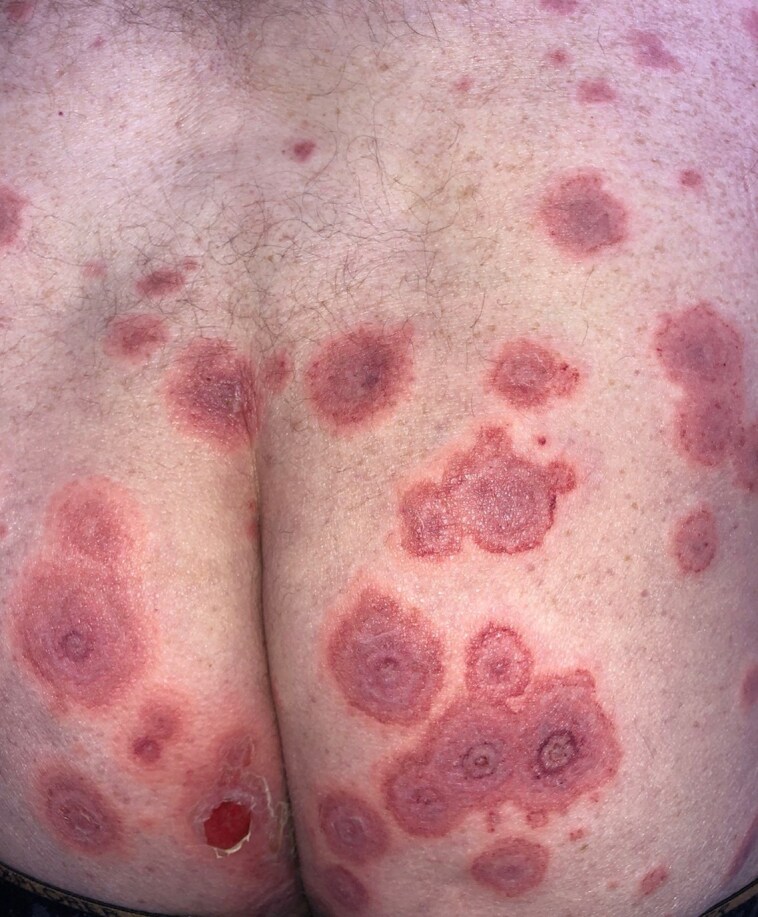
Erythematous, purplish, targetoid-like lesions with purpuric centre maculo-papular rash affecting the lower back. In some places, postblistering erosive lesions can be observed.

**Figure 2:**
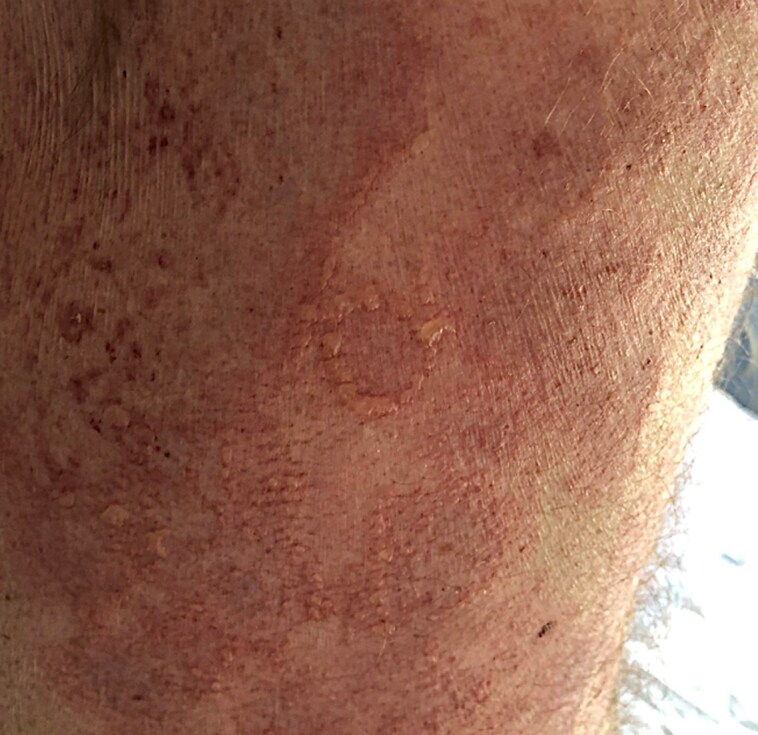
‘String of pearls’ on erythematous skin, circling annular and confluent lesions, observed on the side of the trunk.

**Figure 3:**
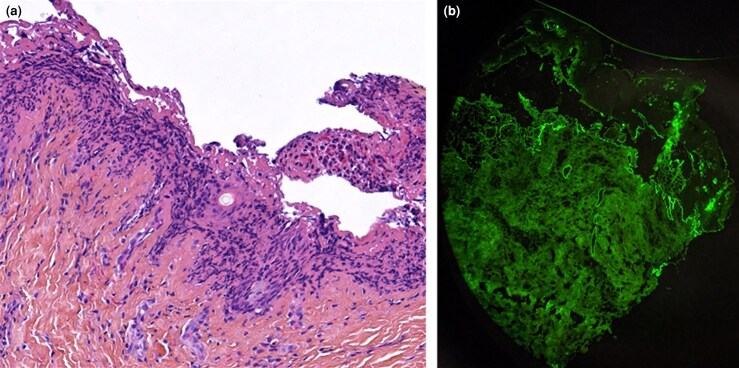
(a) Histological section of lesional skin biopsy showing subepidermal blister with a polymorphic inflammatory infiltrate and neutrophilic microabscesses (haematoxylin and eosin, × 20 magnification). (b) Direct immunofluorescent microscopy of perilesional skin biopsy showing linear deposition of IgA along the dermal–epidermal junction (× 25 magnification).

**Figure 4:**
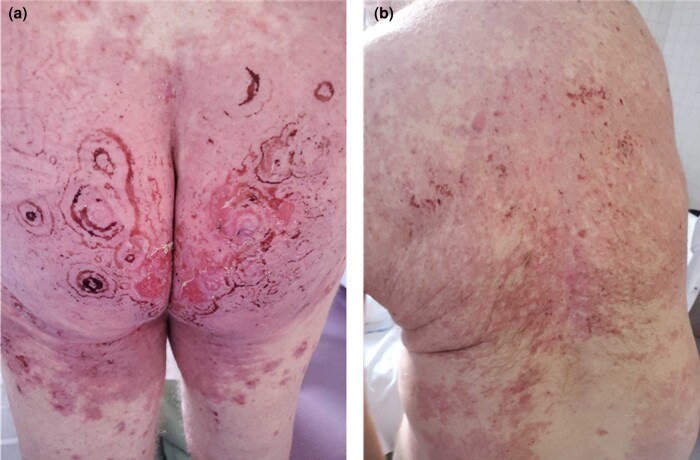
(a, b) Skin lesion improvement 7 days after amoxicillin discontinuation.

### Discussion

LABD is a rare subepithelial autoimmune disease, characterized by linear deposition of IgA at the basement membrane zone. The target antigen is not known precisely but is thought to be LAD285, BP230 and/or BP180, and the NC16A domain or the 120-kDA/97-kDA ectodomain. It is the most common autoimmune blistering disease in children, although it can affect adults; two peaks of onset are noted: during the teenage years and in people’s sixties. A similar case has been described by Lammer *et al*.^2^ They reported the case of a 77-year-old man who presented with multiple tense, fluid-filled blisters, urticarial plaques and targetoid macules with central and perilesional vesicles, and some oral mucosal erosions. Two weeks prior to the rash, their patient was treated with vancomycin. DIF showed linear deposition of IgA along the dermal–epidermal junction, thus confirming LABD. His condition improved a few days after drug discontinuation. LABD can be idiopathic or caused by triggering factors such as drugs.^3^ Vancomycin is the most frequent causal drug (56%), followed by phenytoin (6%) and trimethoprim–sulfamethoxazole (3%).^2^ Amoxicillin-induced LABD is rather rare and involves only 1% of cases of drug-induced LABD. Currently, only a few cases have reported amoxicillin as the culprit drug. Panasiti *et al*.^4^ reported a similar case of a 47-year-old man who developed LABD 7 days after amoxicillin–clavulanate acid intake. This patient had typical rosette-like lesions with purpuric centres that resembled erythema multiforme. Another case reported by Santos-Juanes *et al*.^5^ involved a 67-year-old woman who developed LABD only 24 h after amoxicillin intake, which was much sooner than in our case. Unfortunately, there was no photography provided in the report of Santos-Juanes *et al*. so we could not appreciate the aspect of skin lesions. A final case of amoxicillin-induced LABD involved a 2-year-old child, but the Naranjo score was not provided and therefore the responsibility of amoxicillin in this case is debatable. The eruption typically appears within 9 days (median time) after exposure to the incriminated drug, even if the drug has been discontinued in the meantime. In 75% of patients, drug-induced LABD has a favourable course when the culprit drug is stopped.^6^ In those cases, topical corticosteroids alone can be used to treat the acute phase of the disease, thus avoiding the use of systemic treatment with ensuing side-effects. To date, no clinical difference has been described between idiopathic and drug-induced LABD. It typically manifests as a ‘string of pearls’ pattern, involving the limbs and perioral or ­perigenital skin. Oral and genital mucosa are often involved. Clinical presentation can also be polymorphic, mimicking other autoimmune blistering diseases (bullous pemphigoid, pemphigus vulgaris, mucous membrane pemphigoid), toxic epidermal necrolysis or erythema multiforme.^7^ In a French retrospective pharmacovigilance study, Garel *et al*.^8^ found that 20% of cases (especially those induced by vancomycin) mimicked toxic epidermal necrolysis.

In this case, skin lesions were first identified as atypical erythema multiforme secondary to a bronchopulmonary infection, viral recurrence or amoxicillin. Other differential diagnoses were erythema gyratum repens, erythema annulare centrifugum or autoimmune bullous disease, including LABD. Finally, histopathological and DIF examination established the definitive diagnosis. Nonetheless, we cannot exclude the possibility that this case could be the result of a minor erythema multiforme with IgA-positive DIF, although most positive DIF results appear to show IgM or C3 linear deposition in erythema multiforme.^9^

In conclusion, this case illustrates the polymorphism of drug-induced LABD, here mimicking postinfectious bullous erythema multiforme. Skin biopsy for histopathological and DIF examination should always be performed when bullous lesions are present. Disease management mostly relies on identifying and stopping the culprit drug and starting supportive care.

## Data Availability

The data underlying this article will be shared on reasonable request to the corresponding author.
